# Score for the Overall Survival Probability Scores of Fibrosarcoma Patients after Surgery: A Novel Nomogram-Based Risk Assessment System

**DOI:** 10.1155/2021/4533175

**Published:** 2021-12-22

**Authors:** Yuyuan Chen, Changxing Chi, Dedian Chen, Sanjun Chen, Binbin Yang, Sijia Huang, Zengpai Zheng

**Affiliations:** ^1^Department of Anorectal Surgery, The People's Hospital of Pingyang, Wenzhou, Zhejiang, China; ^2^The Second Department of Breast Surgery, The Third Affiliated Hospital of Kunming Medical University, Kunming, Yunan, China; ^3^Department of Radiation Oncology, The Third Affiliated Hospital of Kunming Medical University, Kunming, Yunan, China; ^4^Department of Pain Treatment, The First People's Hospital of Chenzhou, Chenzhou, Hunan, China; ^5^Wenzhou Medical University, Wenzhou, Zhejiang, China; ^6^Shuitou Town Center Health Center, Wenzhou, Zhejiang, China

## Abstract

**Background:**

The primary purpose of this study was to determine the risk factors affecting overall survival (OS) in patients with fibrosarcoma after surgery and to develop a prognostic nomogram in these patients.

**Methods:**

Data were collected from the Surveillance, Epidemiology, and End Results database on 439 postoperative patients with fibrosarcoma who underwent surgical resection from 2004 to 2015. Independent risk factors were identified by performing Cox regression analysis on the training set, and based on this, a prognostic nomogram was created. The accuracy of the prognostic model in terms of survival was demonstrated by the area under the curve (AUC) of the receiver operating characteristic curves. In addition, the prediction consistency and clinical value of the nomogram were validated by calibration curves and decision curve analysis.

**Results:**

All included patients were divided into a training set (*n* = 308) and a validation set (*n* = 131). Based on univariate and multivariate analyses, we determined that age, race, grade, and historic stage were independent risk factors for overall survival after surgery in patients with fibrosarcoma. The AUC of the receiver operating characteristic curves demonstrated the high predictive accuracy of the prognostic nomogram, while the decision curve analysis revealed the high clinical application of the model. The calibration curves showed good agreement between predicted and observed survival rates.

**Conclusion:**

We developed a new nomogram to estimate 1-year, 3-year, and 5-year OS based on the independent risk factors. The model has good discriminatory performance and calibration ability for predicting the prognosis of patients with fibrosarcoma after surgery.

## 1. Introduction

Fibrosarcoma is a group of malignant soft tissue tumors. As solid tumors composed of fibroblasts, FS often involve the deep soft tissues of the extremities, trunk, head, and neck. Surgery is the primary treatment of fibrosarcoma, but the prognosis of its patients varies depending on the type of tumor [1].

Adult fibrosarcoma used to be the most common soft tissue sarcoma. However, with the increased awareness of soft tissue tumors and fibrosarcoma, the diagnosis of adult fibrosarcoma has been tightly defined so that its incidence may be as low as 1% of adult soft tissue sarcomas [2, 3]. Unlike the highly malignant adult fibrosarcoma, congenital or infantile fibrosarcoma is intermediate in 2020 WHO classification, with few metastases and a better prognosis [4–6]. Since neurotrophic tropomyosin receptor kinase (NTRK) fusion often occurs in infantile fibrosarcoma, the application of NTRK inhibitors such as larotrectinib is highly efficacious in patients [7–9]. Dermatofibrosarcoma protuberans (DFSP) is a low-grade, growing by infiltration but highly locally recurrent fibroblastic malignancy. DFSP most commonly occurs in middle-aged and young adults. Through surgery, radiation therapy, or the targeted drug imatinib mesylate, patients with DFSP have a good prognosis and a high survival rate [10–12]. Surgical resection is the standard treatment for most patients, whether it is a typical or a particular type of fibrosarcoma. Therefore, the evaluation of postoperative prognostic indicators of fibrosarcoma will provide valuable guidance for the clinical treatment of fibrosarcoma.

Nomograms integrate multiple risk indicators and are often used to predict disease survival [13–15]. Fibrosarcoma is primarily treated surgically but has a high recurrence rate after simple resection [2, 16]. Recently, Xiang et al. constructed a nomogram of 663 cases of fibrosarcoma and determined that age, sex, surgical use, tumor stage, pathologic grade, and tumor size may affect cancer-specific survival [15]. However, studies related to predicting overall survival (OS) after surgery in patients with fibrosarcoma have not been reported. This study aimed to develop a nomogram to identify the clinical and pathological factors associated with improved OS in fibrosarcoma patients after surgery using the Surveillance, Epidemiology, and End Results (SEER) database.

## 2. Methods

### 2.1. Inclusion and Exclusion Criteria of Patients

The SEER database is updated annually with the latest cancer information. We identified patients with fibrosarcoma from 2004 to 2015 by the SEER *∗* Stat version 8.3.9. The inclusion criteria were as follows: (1) histology ICD-O-3 was limited to 8810/3, 8812/3, 8813/3, 8814/3, 8823/3, 8832/3, 8833/3, 9321/3, and 9330/3; (2) fibrosarcoma as the patient's primary tumor. The exclusion criteria were as follows: (1) the detailed information lacks age, race, grade, primary site, tumor size, and marital status; (2) unknown historic stage and information related to treatment (radiotherapy and chemotherapy). The final dataset we screened had 439 patients with fibrosarcoma. All patients were randomly divided into a training set (70%) and a validation set (30%) for constructing and validating the nomogram.

### 2.2. Variable Declaration

The age was regrouped as <60 years old (young) and ≥60 years old (old). The race was categorized as white, black, and others. The primary site of the tumor was classified as the head and neck, trunk, and extremities. Tumor size was reclassified as <50 and ≥50 mm. Information on radiotherapy was divided into no radiotherapy, preoperative radiotherapy, and postoperative radiotherapy.

### 2.3. Statistical Analysis

The primary endpoint of this study, overall survival, was analyzed by Kaplan–Meier survival curves, and a log-rank test for significance was performed [14]. Prognostic factors with a *P* value <0.05 in univariate Cox regression analysis were included in multivariate Cox regression analysis. In multivariate Cox regression analysis, *P* values <0.05 were considered statistically significant, indicating that the variables examined were independent risk factors. In addition, hazard ratios and 95% confidence intervals were used to adjust prognostic variables.

Based on the above results, we plotted the nomogram against the training set by the rms package. The discrimination of the nomogram was demonstrated by the area under the curve (AUC) of the time-dependent receiver operating characteristic (ROC) curves. In addition, 1-year, 3-year, and 5-year calibration curves and decision curve analysis (DCA) were generated to validate the nomogram's predictive consistency and clinical value. Through the risk assessment system established by *X*-tile software, the patients with fibrosarcoma after surgery were classified into high-risk, intermediate-risk, and low-risk groups, further demonstrating the application value of the predictive model. All statistics were analyzed by *R* software (version 4.0.3), while *P* <0.05 (two-sided) was considered significant.

## 3. Results

### 3.1. Baseline Characteristics of the Patients

A total of 439 patients with fibrosarcoma after surgery from the SEER database were included according to our criteria. In addition, 308 patients were included in the training set, and 131 patients were included in the validation set. As given in Table 1, the proportion of male patients was higher than that of females (54.5% vs. 45.5%; *P*=0.218). The median age of the primary diagnosis was 52 (25–75%, 37–65). Most patients were white (67.9%), and 65.3% were <60 years old. 7.5% of the population had primary disease sites in the head and neck, 43.2% in the trunk, and 49.4% in the extremities. 35.1% of screened patients with postoperative fibrosarcoma were in stage I, 31.5% in stage II, 17.5% in stage III, and 15.9% in stage IV. 9.7% of the patients received chemotherapy, and 30.5% received radiation therapy, of which 3.6% were preoperative and 26.9% were postoperative. The majority of patients were localized (75.6%), and most tumors were ≥50 mm (55.5%).

### 3.2. Risk Factors in Postoperative Prognosis of Fibrosarcoma

**Figure 1:**
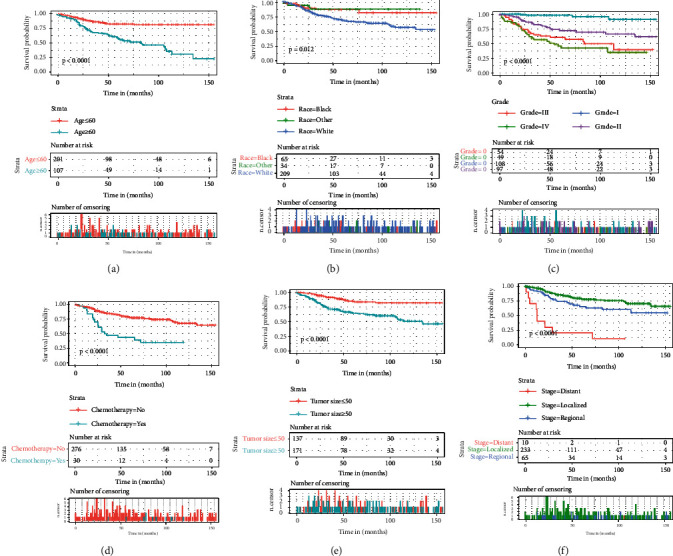
Kaplan–Meier survival curves of fibrosarcoma patients after surgery stratified by (a) age, (b) race, (c) grade, (d) chemotherapy, (e) tumor size, and (f) historic stage.

To identify factors that may predict the occurrence of postoperative fibrosarcoma, univariate and multivariate analyses were performed on the training set. As given in Table 2, the age, race, grade, chemotherapy, tumor size, and historic stage were strongly associated with the OS of the patients with fibrosarcoma after surgery. Consistent with univariate analysis, Kaplan–Meier analysis also showed that clinical factors (age, race, grade, chemotherapy, tumor size, and historic stage) were significantly associated with OS (Figure 1). The multivariate Cox regression analysis confirmed that age, race, grade, and historic stage were independent risk factors (Table 2).

### 3.3. Development and Validation of Nomogram

**Figure 2:**
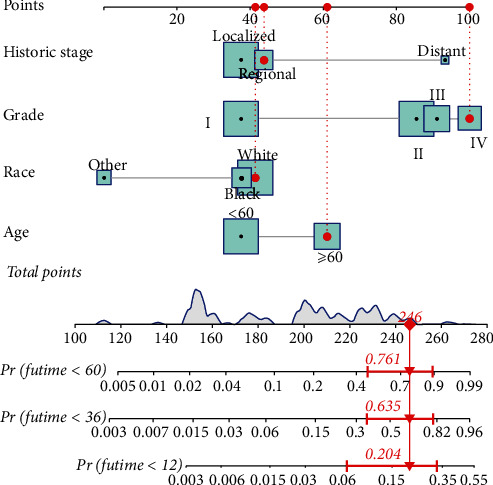
Nomogram for predicting 1-year, 3-year, and 5-year overall survival. The total points were calculated by adding the points of each prognostic factor and correspond to the possibilities of 1-year, 3-year, and 5-year overall survival of fibrosarcoma patients after surgery.

We integrated multiple predictors to make a nomogram based on Cox regression analysis to express the interrelationship between the variables (Figure 2). The tumor grade of fibrosarcoma has the most significant impact on patients, and as tumor grade increases, patients have a worse prognosis. Populations of other races have a better prognosis than those of black or white races. Patients who are ≥60 years old or whose tumors have distant metastases have a worse prognosis.

**Figure 3:**
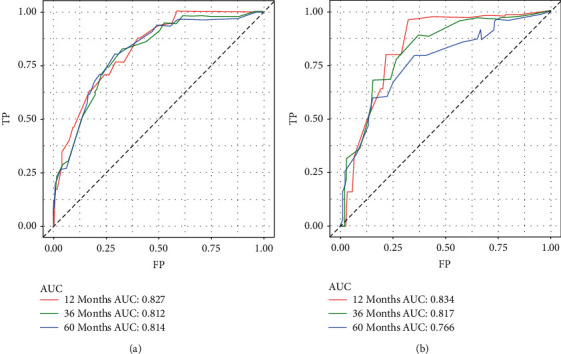
Receiver operating characteristic curves for predicting 1-year, 3-year, and 5-year overall survival in the training set (a). Receiver operating characteristic curves for predicting 1-year, 3-year, and 5-year overall survival in the validation set (b).

**Figure 4:**
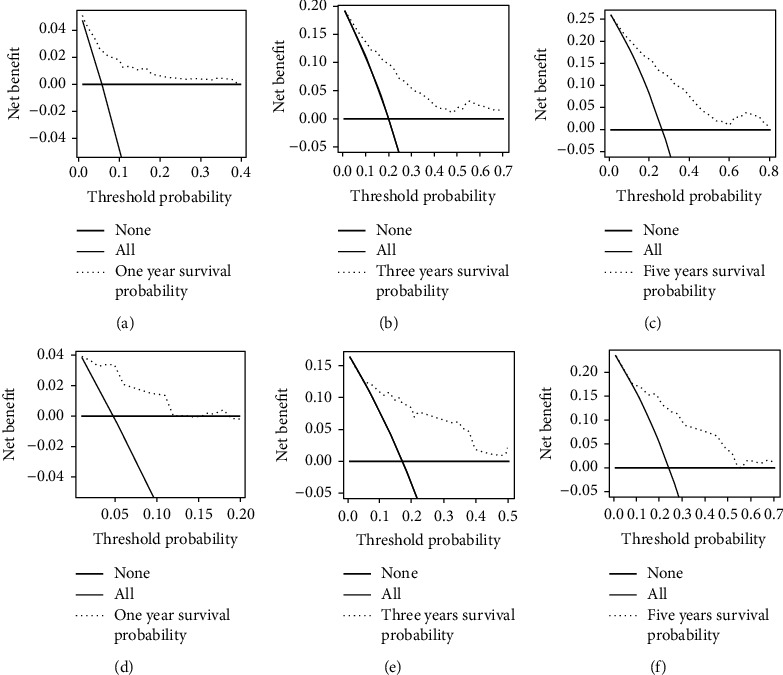
Decision curve analysis of the nomogram for predicting the 1-year (a), 3-year, (b) and 5-year (c) overall survival in the training set and the 1-year (d), 3-year, (e) and 5-year (f) overall survival in the validation set.

**Figure 5:**
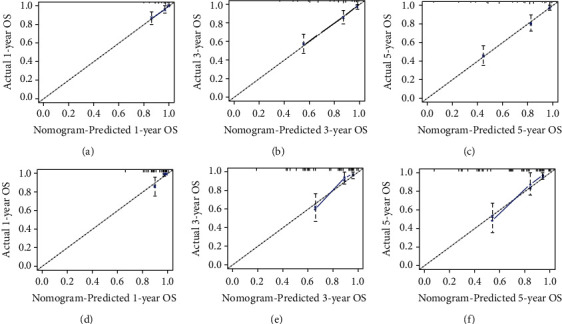
Calibration curves for 1-year (a), 3-year (b), and 5-year (c) prediction in the training set. Calibration curves for the 1-year (d), 3-year, (e), and 5-year (f) prediction in the validation set.

Next, we examine the prognostic models for the training and validation sets, respectively, by plotting ROC curves (Figure 3). The results have demonstrated that the AUC of the training set is 0.827, 0.812, and 0.814 for 12 months, 36 months, and 60 months, respectively (Figure 3(a)), while the validation set is 0.834, 0.817, and 0.766, respectively (Figure 3(b)). The DCA can be used to evaluate the net benefit of nomogram-aided decision-making under different threshold probabilities to assess the benefit degree of patients and the clinical application value of the mode [17]. As shown in Figure 4, this nomogram model has good clinical application in predicting the prognosis of fibrosarcoma after surgery. In addition, the calibration curves for both the training and validation sets have identified a strong agreement between the nomogram prediction and realistic observation for 1-year, 3-year, and 5-year of OS (Figure 5).

### 3.4. Risk Assessment System

**Figure 6:**
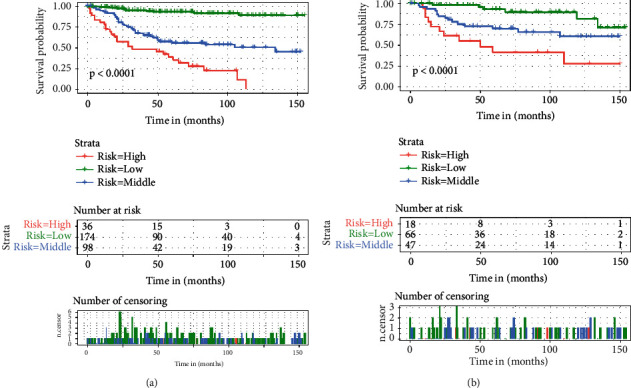
Kaplan–Meier survival analysis for both the training set (a) and the validation set (b).

The total score for each patient was calculated, and all patients were divided into three groups using X-tile software, including high-risk (>231), intermediate-risk (205–231), and low-risk (<205) groups. Kaplan–Meier survival curves for each risk subgroup were plotted, and the results in both the training and validation sets showed differences in OS for patients with different risk levels (*P* < 0.0001) (Figure 6). While patients with high-risk scores had the lowest survival rate, those with low-risk scores had the highest survival rate, indicating that this risk grouping system has a strong predictive value for the prognosis of patients with fibrosarcoma after surgery.

## 4. Discussion

SEER is a typical medical database that provides systematic evidence support and valuable first-hand information for clinicians' practice and medical research [13, 15, 18–20]. Nomogram is conducive to the promotion of personalized medicine and has been proposed to improve disease prediction [21–23]. Wang et al. developed a prognostic nomogram model for small-cell lung cancer patients. They verified that the performance of nomogram was better than that of early models, including those using AJCC staging [24]. As a pictorial representation of a complex mathematical formula, nomograms respond to our exploration of comprehensive biological and clinical models [17, 22, 23].

Fibrosarcoma is a malignant tumor derived from mesenchymal cells, which is usually invasive and has a high postoperative recurrence rate [16]. Adult fibrosarcoma occurs most frequently in the trunk and limbs, followed by the head and neck, consistent with our results [25]. Tumor size of malignant fibrous neoplasms (MFN) of long bones >10 cm is a poor prognostic factor for OS and cancer-specific survival [26]. A report on primary intracranial fibrosarcoma suggested that large tumor volume (≥5 cm) and high Ki-67 index (≥30%) were independent risk factors for OS [27, 28]. Like most types of fibrosarcoma, renal fibrosarcoma is highly malignant and has a poor prognosis. Radical nephrectomy is the primary clinical treatment strategy [29, 30]. Mitotic activity and Ki-67 positive cells were identified as important factors in diagnosing ovarian fibrosarcoma [31, 32]. Less than 5% of fibrosarcoma originated in the genitourinary tract, and fibrosarcoma of the penis is even rarer. To our knowledge, there are few reports on the prognosis of patients with fibrosarcoma. Xiang et al. demonstrated that age, gender, operation, tumor stage, pathological grade, and tumor size might be independent risk factors affecting the survival of FS patients, among which age is the main factor affecting the prognosis [15]. Surgery is the standard treatment for fibrosarcoma at various sites, but the prognosis is abysmal [33]. A reported case of cavernous fibrosarcoma died ten months after the operation, although he received chemotherapy [34]. Patel et al. analyzed 51 sinonasal fibrosarcoma patients from the SEER database. They found that the disease-specific survival rate of patients treated with surgery was better than patients treated with primary radiotherapy alone [35, 36]. However, there has been no evaluation of postoperative prognosis in patients with fibrosarcoma.

This study aims to develop a clinically helpful nomogram that could predict the prognosis of patients with fibrosarcoma after surgery. We finally included 439 patients with postsurgical fibrosarcoma from the SEER database. A randomly divided training set was used to establish the prediction model, while the validation set was used to test the model's accuracy. Through univariate and multivariate analyses, we determined that age, race, tumor grade, and historic stage were closely associated with prognosis (Table 2). We did not find that chemotherapy was an independent prognostic factor for OS, and the results were consistent with a recent study [26]. Based on this, a nomogram was created to facilitate clinical work (Figure 2). Validation is the process of testing models on different groups to obtain unbiased estimates of model performance and judge their applicability to these groups [17]. Knowledge of the identification and calibration of nomograms in this patient group will enable clinicians and patients to comprehensively evaluate the reliability and accuracy of nomograms [17, 37]. Validated by calibration curves, ROC curves, and DCA, our prognostic model showed good internal and external performance in predicting the prognosis of patients with fibrosarcoma after surgery (Figures 3–5).

This study may have limitations due to its retrospective nature. Although the nomogram has good performance, it ignores patients' and doctors' satisfaction and quality of life and lacks clinical utility [17, 38]. Therefore, all nomograms require more validation using large independent groups. Nomograms must be rigorously reviewed before they can be used in clinical decision-making. When we understand the performance and limitations of the predictive model, we can provide a better prognosis for patients.

## 5. Conclusion

In this study, we used the SEER database to analyze the prognosis data of patients with fibrosarcoma after surgery for the first time. Nomograms for estimating OS at 1, 3, and 5 years were established based on a large study cohort. The current model has good prediction ability for patient diagnosis, risk assessment, and clinical decision-making to help clinicians provide highly customized patient management in the future.

## Figures and Tables

**Table 1:** Characteristics of patients with postoperative fibrosarcoma.

Variables	Total set		Training set		Validation set	
	*N* = 439	%	*N* = 308	%	*N* = 131	%
	*n*		*n*		*N*	
Age						
<60	292	66.5	201	65.3	91	69.5
≥60	147	33.5	107	34.7	40	30.5

Race						
Black	92	21	65	21.1	27	20.6
Others	40	9.1	34	11	6	4.6
White	307	69.9	209	67.9	98	74.8

Sex						
Female	201	45.8	140	45.5	61	46.6
Male	238	54.2	168	54.5	70	53.4

Marital status						
Married	220	50.1	151	49	69	52.7
Unmarried	219	49.9	157	51	62	47.3

Primary site						
Head and neck	39	8.9	23	7.5	16	12.2
Trunk	191	43.5	133	43.2	58	44.3
Extremity	209	47.6	152	49.4	57	43.5

Grade						
I	142	32.3	108	35.1	34	26
II	133	30.3	97	31.5	36	27.5
III	86	19.6	54	17.5	32	24.4
IV	78	17.8	49	15.9	29	22.1

Radiotherapy						
No	302	68.8	214	69.5	88	67.2
RPS	14	3.2	11	3.6	3	2.3
RAS	123	28	83	26.9	40	30.5

Chemotherapy						
No	389	88.6	278	90.3	111	84.7
Yes	50	11.4	30	9.7	20	15.3

Tumor size						
<50	200	45.6	137	44.5	63	48.1
≥50	239	54.4	171	55.5	68	51.9

Historic stage						
Localized	334	76.1	233	75.6	101	77.1
Regional	89	20.3	65	21.1	24	18.3
Distant	16	3.6	10	3.2	6	4.6

RPS, radiation prior to surgery; RAS, radiation after surgery.

**Table 2:** Univariate and multivariate Cox regression analyses of patients with postoperative fibrosarcoma.

Characteristics	Univariate analysis		Multivariate analysis	
	HR (95% CI)	*P* value	HR (95% CI)	*P* value
Age				
<60	Reference		Reference	
≥60	3.531 (2.236–5.575)	≤0.001	2.776 (1.688–4.566)	≤0.001

Race				
Black	Reference		Reference	
Others	0.789 (0.238–2.622)	0.699	0.209 (0.051–0.851)	0.029
White	2.246 (1.079–4.678)	0.031	1.161 (0.524–2.571)	0.712

Sex				
Female	Reference			
Male	1.327 (0.846–2.081)	0.218		

Marital status				
Married	Reference			
Unmarried	1.295 (0.828–2.026)	0.257		

Primary site				
Head and neck	Reference			
Trunk	0.723 (0.314–1.667)	0.447		
Extremity	1.083 (0.489–2.400)	0.844		

Grade				
I	Reference		Reference	
II	7.157 (2.490–20.574)	≤0.001	8.068 (2.756–23.621)	≤0.001
III	14.008 (4.859–40.386)	≤0.001	10.298 (3.520–30.130)	≤0.001
IV	18.089 (6.311–51.847)	≤0.001	14.840 (5.060–43.521)	≤0.001

Radiotherapy				
No	Reference			
Radiation prior to surgery	1.438 (0.518–3.992)	0.485		
Radiation after surgery	1.295 (0.808–2.077)	0.283		

Chemotherapy				
No	Reference		Reference	
Yes	3.429 (2.041–5.760)	≤0.001	0.879 (0.440–1.757)	0.715

Tumor size				
<50	Reference		Reference	
≥50	3.050 (1.802–5.160)	≤0.001	1.597 (0.904–2.821)	0.107

Historic stage				
Localized	Reference		Reference	
Regional	1.731 (1.051–2.851)	0.031	1.197 (0.712–2.010)	0.498
Distant	9.271 (4.513–19.047)	≤0.001	10.556 (3.857–28.889)	≤0.001

## Data Availability

The dataset from the SEER database that was generated and/or analyzed during the current study is available in the SEER dataset repository (https://seer.cancer.gov/).

## Abbreviations

NTRK: Neurotrophic tropomyosin receptor kinase
DFSP: Dermatofibrosarcoma protuberans
OS: Overall survival
SEER: Surveillance, Epidemiology, and End Results
AUC: Area under the curve
ROC: Receiver operating characteristic
DCA: Decision curve analysis.
